# Male reproductive system and spermatogenesis of *Limodromus assimilis* (Paykull 1790)

**DOI:** 10.1371/journal.pone.0180492

**Published:** 2017-07-19

**Authors:** Lea F. Schubert, Stephanie Krüger, Gerald B. Moritz, Veit Schubert

**Affiliations:** 1 Martin-Luther-University Halle-Wittenberg, Halle, Germany; 2 Leibniz Institute of Plant Genetics and Crop Plant Research (IPK) Gatersleben, Seeland, Germany; University of Hyderabad, INDIA

## Abstract

Based on advanced light and electron microscopy, we describe the male reproductive system and sperm development of *Limodromus assimilis*. The genital tract consists of pairs of uni-follicular testes, spermatic ducts with diverticula regions, seminal vesicles, accessory glands, an unpaired ejaculatory duct and an aedeagus containing an internal sac equipped with sclerotic scales. Based on their morphology, we draw conclusions about their functions. After spermatogenesis within the follicle, the spermatozoa become released from the sperm cysts. The single spermatozoa move into the diverticula of the vasa deferentia I. Here, they become attached to central rods (spermatostyles), forming secondary conjugates (spermiozeugmata). The coordinated flagella movement of the conjugates possibly improves sperm velocity. Using super-resolution microscopy, we identified highly condensed reticulate chromatin in the lancet-shaped spermatozoa heads and the mitochondrial derivates of the flagella, likely formed by genomic and mitochondrial DNA, respectively. The results show, for the first time, sperm bundle formation in a Platynini species mainly corresponding to that found in Pterostichini species.

## Introduction

Insect reproductive systems show large morphological variability. Similarly, their spermatozoa may vary strongly in shape and size [1, 2]. In addition to single flagellate and multi-flagellate spermatozoa, others without flagella appear [3]. Some species form conjugates of spermatozoa, first identified by **Gilson** [4]. These were described in orders such as Odonata [5], Hymenoptera [6], Orthoptera [7] and Coleoptera [8].

Within the Carabidae, spermatozoa morphology has already been analysed in the taxa Cicindelinae [9], Scaritinae [10], Carabinae [11, 12], Pterostichini [13–17] and Platynini [18]. Cicindelidae and Scaritinae species do not form sperm bundles (spermiozeugmata). In the three other taxa the spermatozoa heads are embedded in a hyaline carrier structure called a spermatostyle, central rod, carrier rod or cap. These structures may vary significantly in size, shape and number of spermatozoa included, even between closely related species.

Within the *Pterostichini*, the flagella are completely movable within the conjugate, or they are attached to the spermatostyle [17]. In some species, the spermatostyles can be longer than the spermatozoa [14], and they are able to form spirals, such as in *Abax parallelpipidus* Piller et Mitterpacher 1783 (Carabidae: Pterostichini), producing a 17-times twisted spiral formed by a central spermatostyle where spermatozoa are attached [19]. Short, rod-like spermatostyles were also described in this tribe. Between different species, the length varies from 0.11 to 25.3 mm [17]. *Carabus insulicola* Chaudoir 1869 (Carabinae), endemic to Japan, forms heteromorphic sperm conjugates in which the spermatostyles, where spermatozoa associate, are of varying lengths [11, 12]. Until now, only in one Platynini species, *Jujiroa estriata* Sasakawa, spermiozeugmata were analysed. This species produces 2.4-mm long bundles containing movable flagella [18].

The reason some species form sperm bundles is still under debate. They seem to be essential for increasing the sperm transfer efficiency to the female during copulation. It is thought that spermatozoa aggregation improves their mobility compared to that of single spermatozoa [20, 21]. In addition, physical protection from spermicidal environments [22] and improved egg penetration [20] have been discussed as possible advantages. Less is known about sperm bundle formation. Thus, analysing the morphology of the male reproductive organs is required.

Each of the twin testis of Adephaga (Coleoptera), thus also of Carabidae, is formed by only one raised follicle [23] containing sperm cysts where spermatogenesis proceeds [24]. Vas efferens and vas deferens, parts of the spermatic duct, are attached posteriorly. In general, insect vasa deferentia are undifferentiated tubes enlarged at their posterior ends forming seminal vesicles (vesiculae seminales) [24, 25]. However, the vasa deferentia of Carabidae species differentiate into excrescences [19], called lobes [13], bursas [19] or diverticula [14]. Light and electron microscopical investigations of Pterostichini species, such as *A*. *parallelpipidus* and *Pterostichus nigrita* (Paykull 1970), indicate that in these structures, the formation of spermiozeugmata appears [14, 19, 26]. **Higginson and Pitnick** [21] described these as secondary sperm conjugates because they are formed in the post-follicular parts of the genital tract. In contrast, primary conjugates (spermatodesmata) already appear during the spermatogenesis in the follicle, in which the spermatozoa of a sperm cyst form the complete conjugate.

In some conjugate-forming Carabidae species, the mature spermatozoa are released from the sperm cysts, are transferred via the short vas efferens into the diverticulum of the vas deferens region, and are attached here to the spermatostyle. The spermatostyle becomes secreted from the diverticulum epithelium, and after reaching a defined length, it becomes released from the diverticula. Then, spermiozeugmata are transported to the seminal vesicle to be stored until copulation [14, 19, 26].

The chemical composition of Carabidae spermatostyles is not yet clear. The double spermatozoa of *Colymbetes fuscus* (Linnaeus 1758) (Coleoptera: Dytiscidae) are connected via proteins and polysaccharides [27]. Proteins were also proven to be part of the spermatostyles and conjugation substance of *Parachauliodes japonicus* (McLachlan 1867) (Megaloptera: Corydalidae) and *P*. *nigrita* (Carabidae) [28, 29].

Accessory glands, also belonging to the male reproductive system, may be present in different numbers and shapes in Coleoptera species [25]. In Harpalinae (Coleoptera) species, **Will et al**. [30] found two curved accessory glands. In general, accessory glands produce a viscous exudate required to form the spermatophore, representing a structure composed of spermatozoa and a secretion, which becomes transferred into the female via copulation [24]. The main components of the gland secretion are polysaccharides, proteins and mucosubstances [31]. In most insects’ posterior, the accessory glands fuse and form the ejaculatory duct laying in the tube of the copulation apparatus called the aedeagus. This morphologically species-specific organ contains the protrusive internal sac (endophallus), which may be equipped with special tiles and spines [24].

**Fig 1:**
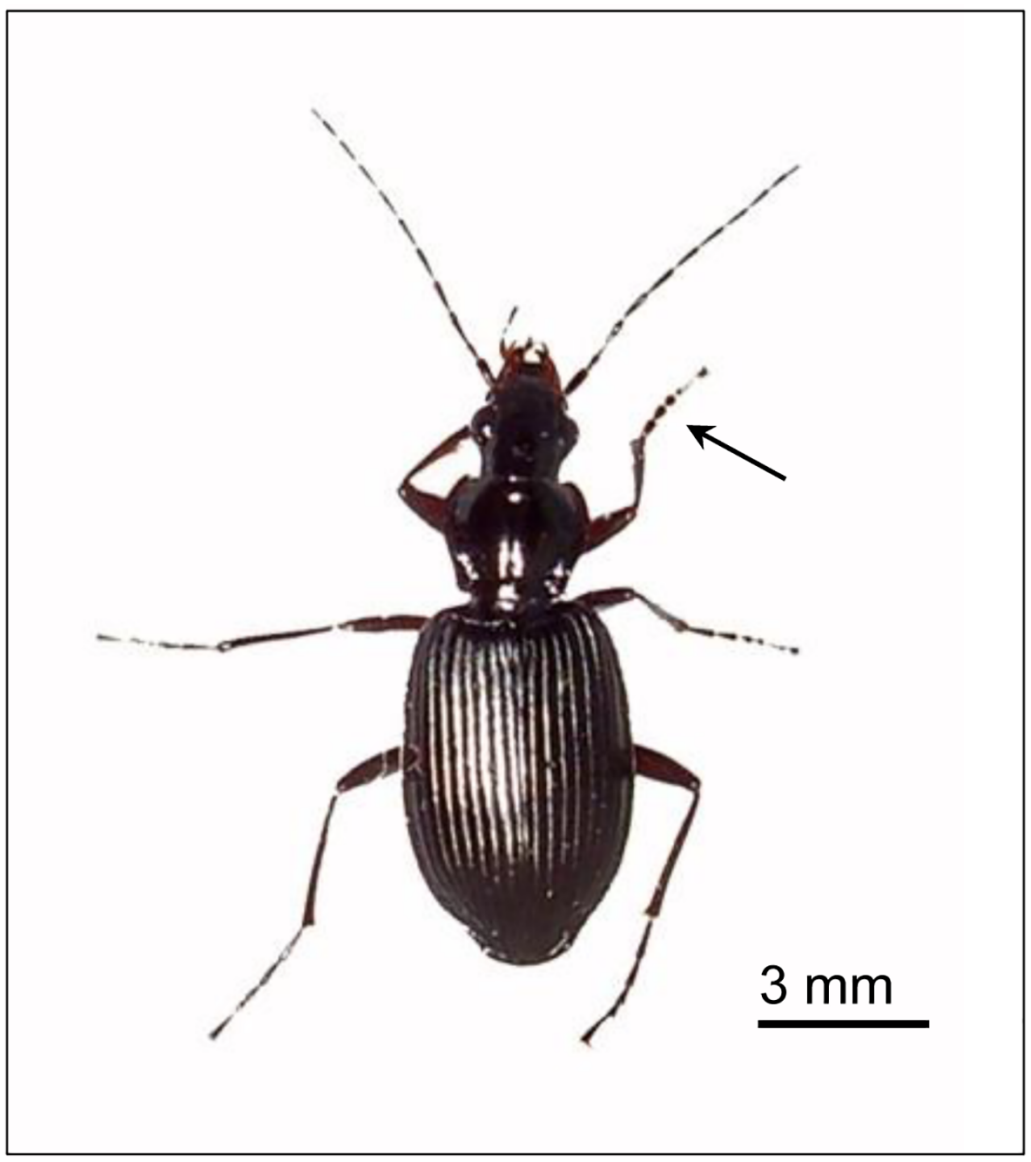
Male imago of *L*. *assimilis*. The wide tarsen (arrow) allows one to distinguish males from females.

Previously, the morphology and formation of Carabidae spermiozeugmata were mainly described within the tribe Pterostichini originating from East Asia [17]. It was not yet clear whether the formation of spermiozeugmata, as described for Pterostichini, is also true for other Carabidae species. Therefore, we analysed *Limodromus assimilis* (Paykull 1790), which belongs to the Platynini tribe also containing sperm bundles [18]. Due to its eurytopicity, the black 10- to 12-mm-long ground beetle (Fig 1) is common, in addition to many other Palaearctic areas, in riparian forests of Middle Europe [32]. The propagation of hibernated imagos occurs during spring.

In the present work, we describe the morphology of the male reproductive system, spermiozeugmata formation and the spermatogenesis of *L*. *assimilis*. Based on these observations and simple staining procedures delivering first rough hints on the chemical components of the reproductive organs and tissues, we conclude their functions.

## Materials and methods

### Origin and animal propagation

Male *L*. *assimilis* individuals (Fig 1) were captured April 2012 in live traps at two different sites in Germany: the nature reserve Burgholz, Halle (Saale) (51°25`01.89” N, 11°59`41.84”O) and Landsberg (Saalekreis) (51°33`51.50”N, 12°07`58.37”O). Captures were carried out with permission of the Saxony-Anhalt Regional Office for Environmental Protection (LAU).

Until preparation, the beetles were kept in a climate chamber (Sanyo) under long-day conditions (16 h light, 8 h dark, 20000 Lux) at 23°C in order to positively influence the maturation of the genital organs. *Tenebrio molitor* Linnaeus 1758 larvae served as food.

### Sample preparation and staining procedures

The beetles were killed with chloroform, and the dissected organs were put into Ringer’s solution to remove the trachea, Malpighian tubules and fat bodies. Spermiozeugmata were obtained by scratching out the seminal vesicles in Ringer’s solution. The genital organs were fixed in Carnoy’s solution, treated in an ascending ethanol series (70%, 80%, 96%) and isopropanol, and imbued with paraffin at 65°C. Using a Leica (SM 2010 R) microtome, the sample blocks were cut in 5- or 8-μm thin sections and then placed onto protein-glycerine treated slides. The deparaffinised samples were stained with haematoxylin and eosin (HE) after **Romeis** [33].

The aldehyde fuchsin-alcian blue (AF-AB) staining was applied after **Spicer and Meyer** [34]. For the alcian blue and periodic acid-Schiff (AB-PAS) staining, the samples were first treated with alcian blue, followed by periodic acid, Schiff’s reagent and haematoxylin. Then, the sections were embedded in Canada balsam. For DAPI staining the spermiozeugmata were placed onto polysine coated slides (Thermo Scientific) into a drop of Ringer’s solution. Then, 8 μl of 0.005% DAPI in Antifade (Vectashield) was added, and the sample was covered by an 18×18 mm coverslip.

### Light microscopy

The genital organ sections and the DAPI-stained spermiozeugmata were analysed under a Leica microscope (Leitz DMRBE) applying Differential Interference Contrast (DIC) and fluorescence microscopy, respectively. To analyse the structure of chromatin in spermatozoa heads and the mitochondrial derivates above the diffraction limit of light (super-resolution), spatial Structured Illumination Microscopy (3D-SIM) was applied using a C-Apo 63×/1.2W Korr objective of an Elyra PS.1 microscope system and the software ZEN (Carl Zeiss GmbH) to achieve a lateral resolution of ~120 nm and an axial resolution of ~250 nm. Images were captured using a 405-nm laser for excitation and the appropriate emission filter to identify DAPI [35]. SIM image stacks were used to produce 3D movies by Imaris 8.0 (Bitplane) and ZEN software.

### Scanning electron microscopy

The evaginated aedeagus was fixed in Carnoy’s solution, dehydrated with ethanol (70%, 80%, 96%) and isopropanol and then fixed in hexamethyldisilazane according to Bock [36]. The dry samples, glued on a plate, were coated with gold and then investigated with a Hitachi (S-2004) scanning electron microscope at 18 kV.

## Results

### Morphology of the male reproductive system

**Fig 2:**
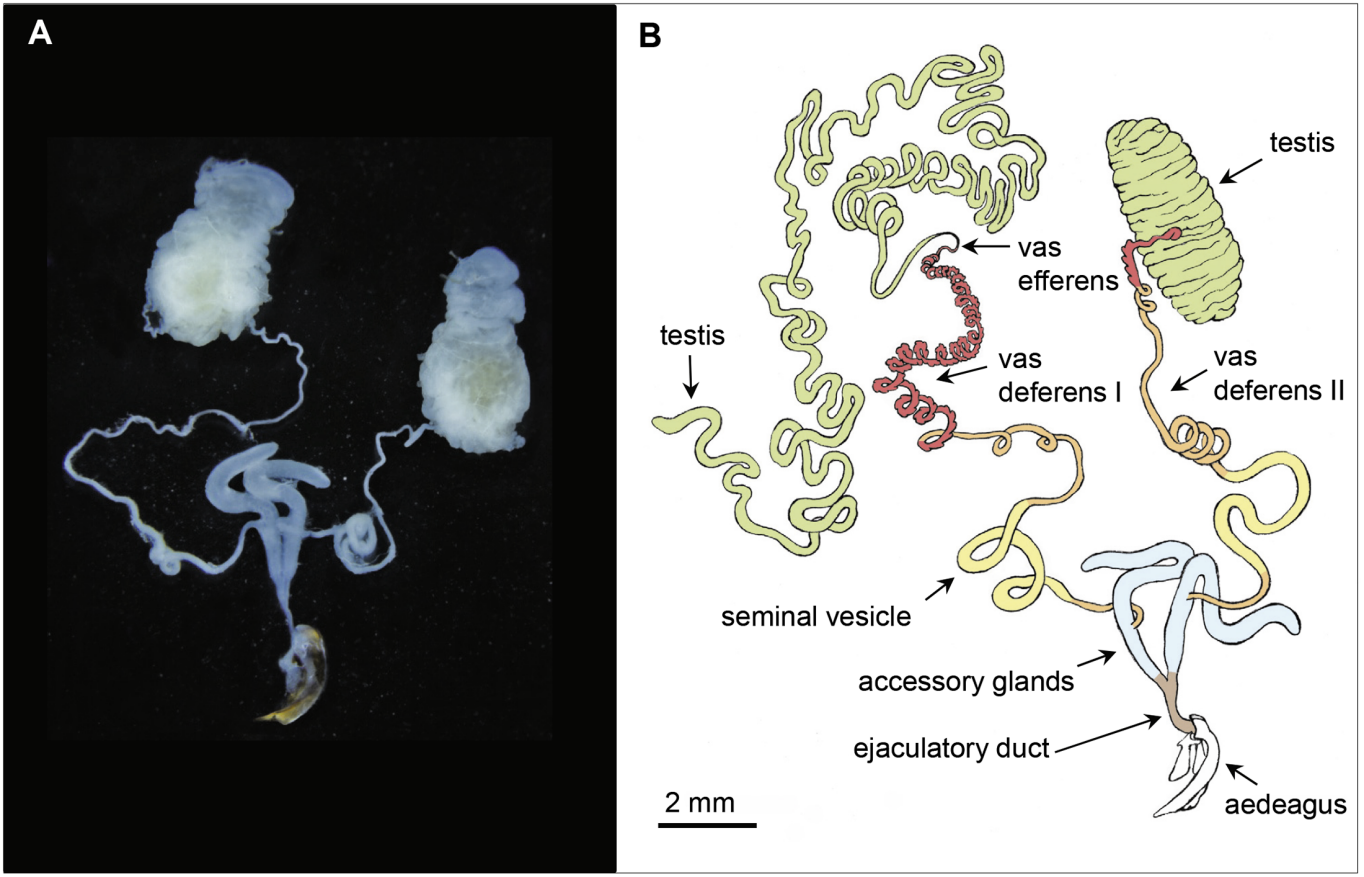
Organs of the male reproductive system. **(A)** The different colours in the scheme **(B)** where the left testis is shown untwisted indicate the main functional parts.

Fig 2 provides an overview of the male reproductive system of *L*. *assimilis* and S1 Table summarizes size measurement data of its main parts. Testes, spermatic ducts and accessory glands are present in pairs. Testes are localized lateral-dorsal and the accessory glands median-ventral in the abdomen. The globular and unifollicular testes have a size of ~3.8 x 1.9 mm comprising one follicle, the spermatic duct and the vas deferens-region I.

First, the follicle leads into a short vas efferens (defined by **Snodgrass** [24]) followed by the three parts of the vas deferens. The first spiral part (vas deferens I) contains several diverticula. The tube is twisted either left-handed, right-handed or alternating left- and right-handed, at which the loops are extended in the posterior direction. The second part (vas deferens II) does not contain diverticula, and the third part, known as seminal vesicle (defined by **Kaulenas** [25] and **Snodgrass** [24]), represents a part of the spermatic duct with an increased diameter. Eventually, the spermatic duct leads into the posterior third part of the accessory glands. The measured length of the whole spermatic duct from the follicle to its outlet averages ~118 mm. The curved accessory glands re-join posterior into the ejaculatory duct which leads into the median lobe of the aedeagus. The invaginated median lobe lies within the abdomen at the side, in which its tip points to the left when regarded dorsal-caudally. In addition, the aedeagus has two lateral lobi, called parameres.

### Spermatogenesis

**Fig 3:**
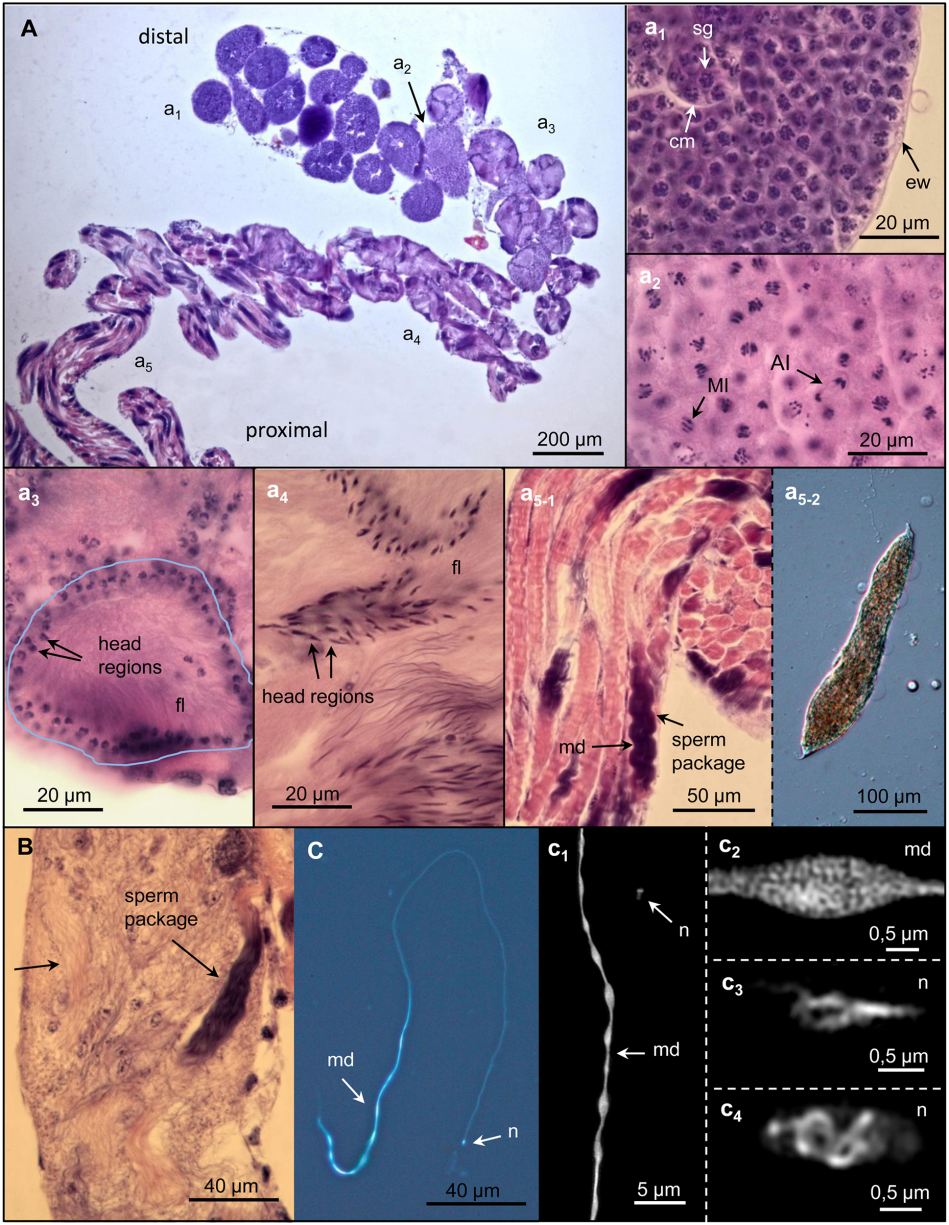
Spermatogenesis stages in the testis. **(A)** Testis section showing different stages of sperm development after haematoxylin-eosin (HE) staining. **(a**_**1**_**)** The young part of a follicle surrounded by the epithilial wall (ew) contains sperm cysts with spermatogonia (sg) inside. The spermatogonia enclosed by sperm cyst membranes (cm) undergo several mitoses. **(a**_**2**_**)** Spermatogonia during meiosis. Metaphase I (MI) and Anaphase I (AI) cells are marked. **(a**_**3**_**)** Early spermatids with globular nuclei in the head and short flagella (fl). The sperm heads are disposed to the cyst membrane (labelled by the blue line). **(a**_**4**_**)** Advanced spermatid stage. The mitochondrial chromatin becomes stretched along the elongated flagella. **(a**_**5-1**_**)** Final stage of spermatogenesis with fully elongated spermatozoa. Within a cyst (sperm package) spermatozoa are parallel to each other. A fixed flagellar movement is visible, in which the mitochondrial derivates (md) appear darkly stained by HE. **(a**_**5-2**_**)** Sperm package extracted from the posterior region of the testis. **(B)** Dissipation of sperm packages (arrow) by dissolution of the sperm cyst membranes in the posterior part of the testis. **(C)** Mature spermatozoon stained by the DNA specific dye DAPI. SIM reveals the chromatin structure of the mitochondrial derivates (md) **(c1, c2)** and of the nucleus (n) in lateral **(c3)** and plan **(c4)** view.

The formation of spermatozoa occurs in the testis follicles, which are divided into different parts of progressive spermatozoa development within sperm cysts (Fig 3A). The follicle is surrounded by a thin epithelial layer and the diameter of its distal part reaches ~170 μm. In this region, the spermatogonia are localized and originated from primordial germ cells via mitosis. Depending on the different number of already-performed somatic divisions, a different number of these are enclosed within a sperm cyst (Fig 3a_1_). A thin epithelial cell layer surrounds the sperm cyst. The continuous spermatogonia generation induces their proximal movement. After mitosis completion, the spermatogonia nearly double in size and then perform meiosis as indicated by different stages (Fig 3a_2_). The resulting globular spermatids elongate during spermatogenesis starting with flagella formation, in which for now the chromatin (shown in violet by HE staining) remains in the globular sperm head (Fig 3a_3_). Afterwards, the chromatin becomes elongated, indicating the mitochondrial derivate formation along the flagella. The spermatid heads are disposed to the cyst membrane, where they seem to be attached (Fig 3a_4_). Later, in the head, the nucleus containing condensed chromatin becomes visible (Fig 3C). In this state the flagella are arranged nearly straight to each other. Afterwards, they show a synchronous wave-like behaviour suggesting that they have reached their functionality. In this spermatogenesis state, the spermatozoa reached their final lengths and lie tightly within the sperm cysts. Via HE staining, the mitochondrial derivates in the flagella become visible (Fig 3a_5-1_ and 3a_5-2_). Here, the follicle diameter amounts to 160 μm and then narrows in the region ahead of the vas efferens where the sperm cyst membranes dissolve, and the spermatozoa are released (Fig 3B). The vas efferens, showing a diameter of ~50 μm, exhibits a muscle layer and a broad layer of epithelial cells. The separated spermatozoa, showing a mean length of ~310 nm, are transported via this short spermatic duct region into the following vas deferens I.

To analyse the distribution and structure of chromatin within spermatozoa, they were labelled by the DNA-specific stain DAPI followed by super-resolution microscopy (SIM). The highly condensed genomic chromatin was identified in the spearhead-like shaped sperm heads. In addition, chromatin-free regions are evident in the sperm head nucleus (Fig 3c_3-4_, S1 and S2 Movies). The mitochondrial DNA of mature spermatozoa forming two parallel connected mitochondrial derivates of the same shape and size was present posteriorly within the second half of the flagella region and extended ~80–100 μm. At a distance of ~5 μm, the derivates exhibited narrow constrictions of ~300 nm in diameter (Fig 3c_1-2_, S1 Fig, S3 and S4 Movies).

### Sperm bundle formation and transport

**Fig 4:**
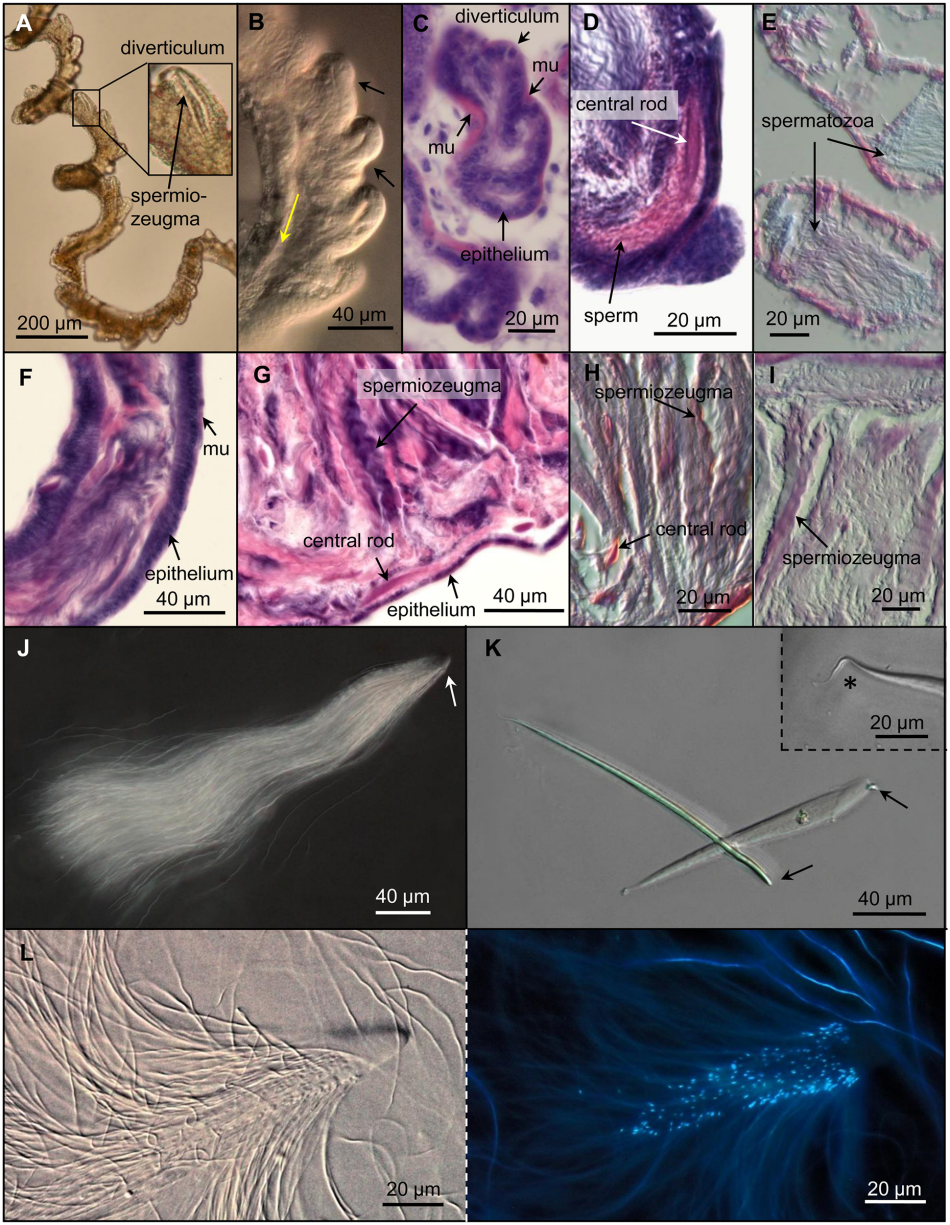
Sperm bundle formation in vasa deferentia I (A-E), their transport in vas deferens II (F) and seminal vesicle (F, G), and the structures of spermiozeugmata (J-L). **(A)** Part of vas deferens I with diverticula, each containing a spermiozeugma (true colour bright field microscopy). **(B)** Part of vas deferens I with differently contracted diverticula (black arrows). The direction of the sperm bundle transport is marked by the yellow arrow (DIC). **(C, D)** Sections of vas deferens I stained with HE show diverticula with a cuboidal epithelium surrounded by a muscular layer (mu) **(C)** and a spermiozeugma located within a diverticulum consisting of a central rod and attached spermatozoa **(D). (E)** Oblique section of vas deferens I. AF-AB staining shows blue- and magenta-coloured sperm. **(F)** Longitudinal section of a part of vas deferens II without diverticula (HE stained) showing a columnar epithelium and a thin muscular layer (mu). **(G)** Longitudinal section of seminal vesicle (HE stained) filled with spermiozeugma. The squamous epithelium is marked. **(H)** Longitudinal section of the seminal vesicle. AB-PAS staining shows spermatozoa in magenta and central rods blue-red coloured. **(I)** Longitudinal section of the seminal vesicle. AF-AB staining shows magenta coloured spermiozeugmata. **(J-L)** Spermiozeugma isolated from the seminal vesicle. **(J)** Many spermatozoa are attached to the apical part of the central rod (arrow) of a spermiozeugma (darkfield microscopy). **(K)** Lateral and plan view of central rods. The apical parts are marked by arrows, the tail piece (inset) by an asterisk (DIC). **(L)** Anterior part of a spermiozeugma shown in DIC (left) and DNA specific (DAPI) fluorescence (right). The DNA labelling of the nuclei allows to quantify the number of spermatozoa within a sperm bundle.

The first (I) region of the vasa deferentia appears as an ~20-mm long spiral containing more than 400 diverticula. All these wall protrusions are outside of the spiral and their tips are oriented towards the vas efferens (Fig 4A and 4B, S5 Movie). The anterior part of vas deferens I close to vas efferens (S2 Fig) has a diameter of ~28 μm and comprises smaller ovoid-like diverticula (~27 x 22 μm) than in the posterior part where the diameter increases up to 60 μm, and the thumb-shaped diverticula reach a length and width of ~70 μm and ~50 μm, respectively. The vas deferens I wall consists of a cuboidal epithelium surrounded by a muscular layer. The HE staining indicates DNA (Fig 4C). In this part of the spermatic duct, spemiozeugmata appear first. The lumen of each diverticulum contains a single central rod and the anterior parts of the spermatozoa (Fig 4D). Life observations indicated that the rhythmic contractions of the diverticula are accompanied by a lateral wall constriction and consequently by a lumen reduction (S5 Movie). The diverticula opening orients towards the seminal vesicle (Fig 4B), and in the same direction, the completive sperm conjugates are transported via peristaltic movements of the spermatic duct.

The spermiozeugmata comprise a central rod where up to ~200 spermatozoa are attached. Their mean length is ~335 μm (Fig 4J–4L, S1 Table). The slightly bent central rod has a length of ~160 μm and can be subdivided into a head, middle and tail part (Fig 4K). The convex head region showing a rounded apex ~23 μm long. The middle piece (width: ~13–15 μm; height: ~24 μm) narrows towards the short filamentary tail. The red central rod stained by AB-PAS reagent indicates the presence of polysaccharides (Fig 4H). The spermatozoa attach to the front two-thirds of the central rod via their heads (Fig 4L). Living spermatozoa, isolated from the seminal vesicle, show a coordinated sinusoidal cilium-like flagella movement. This results in a spiral movement allowing the sperm conjugates to move in Ringer’s solution ~200 μm/min (S6 Movie).

The second vas deferens region (II) does not contain diverticula, has a diameter of ~70 μm and is only slightly contorted. HE staining indicated that it consists of a single-layer columnar epithelium surrounded by a thin muscle layer (Fig 4F). Similar to vas deferens I, the sperm bundles are transported via propulsive peristaltic movements of the spermatic duct.

The seminal vesicle is an ~4–6 mm long part of the vasa deferentia and has, depending on its filling state, a variable diameter of ~150–250 μm. Similar to vas deferens II, the wall consists of a single-layer columnar epithelium surrounded by a thin muscular layer. The seminal vesicle accumulates and stores a high amount of spermiozeugmata (Fig 4G). After AB-PAS staining, the spermatozoa show a mixed colour of magenta and blue, indicating the presence of acid and neutral mucosubstances (Fig 4H). An AF-AB staining leading to an exclusive magenta labelling of the spermiozeugmata suggests the presence of sulphured mucosubstances (Fig 4I). In contrast, spermatozoa present in the spermatic duct region between the proximal follicle and vas deferens I show both magenta and blue. Thus, it seems that they contain in addition carboxylated glycoproteins (Fig 4E).

**Fig 5:**
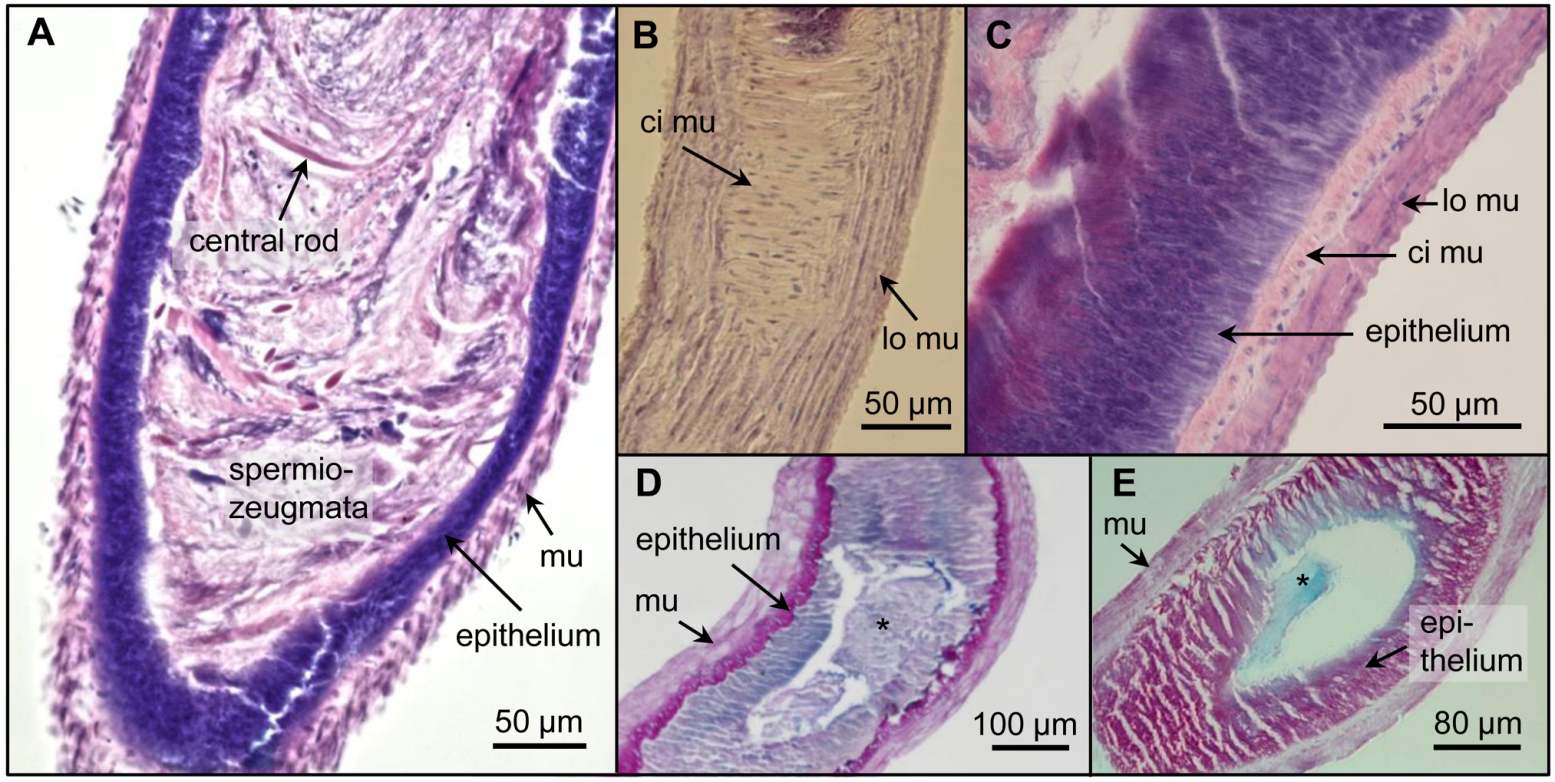
Longitudinal sections of accessory glands stained with HE (A–C), AB-PAS (D) and AF-AB (E). **(A)** Posterior part of an accessory gland filled with numerous spermiozeugmata. **(B)** Circular (ci mu) and longitudinal (lo mu) muscular layers are visible in the surface section. **(C)** The columnar gland wall epithelium is surrounded by circular and longitudinal muscle layers. **(D)** The secretion (asterisk) in the glandular lumen shows a mixed colour of blue and magenta indicating acid and neutral glycoproteins, polysaccharides, glyco- and phospholipids. **(E)** The blue colour of the secretion (asterisk) indicates that the acid glycoproteins yield carboxyl groups.

From the seminal vesicle, the sperm bundles are transported to the accessory glands via a tight corridor which has a strong circular muscular layer. The accessory glands (length: ~4–6 mm, width: ~350–400 μm) are filled with a white viscous secretion. The inner wall epithelium (~12 μm thick) contains secretory adenocytes. This columnar gland wall epithelium is surrounded by circular and longitudinal muscle layers ~30 μm thick (Fig 5A–5C). An AB-PAS and AF-AB staining of the glandular lumen indicates acid and neutral glycoproteins, polysaccharides, and glyco- and phospho-lipids. The blue colour of the secretion specifies that the acid glycoproteins yield carboxyl groups (Fig 5D and 5E).

**Fig 6:**
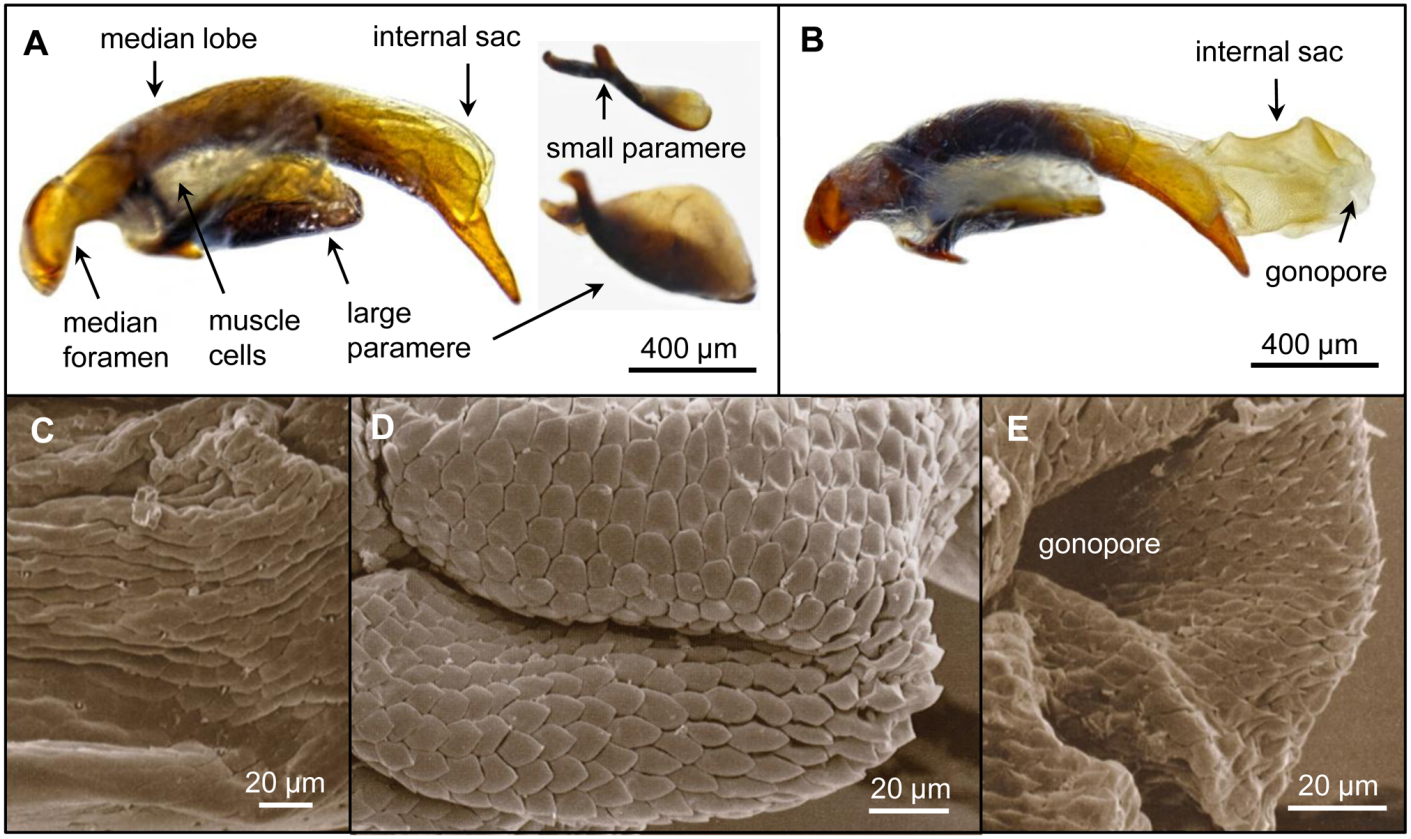
**(A)** Aedeagus (lateral view) shown by light (A, B) and its internal sac structures by electron microscopy (C–E). Aedeagus with internal sac invaginated and **(B)** evaginated. Both parameres are also shown separately in **(A)**. **(C)** Basal region of the internal sac with flat scales. **(D)** Median region of the internal sac with more or less taper scales. **(E)** Apical region of the internal sac with spicule-like scales.

Sperm bundles present in the accessory glands are transported via contraction of their double-layered musculature (Fig 5A) into the ejaculatory duct. The area where the accessory glands fuse (diameter: ~230 μm) contains a sclerotic structure dividing them (S3 Fig). The ejaculatory duct is also surrounded by muscle cells mediating the sperm bundle transport via contraction. Its end flows into the median foramen of the median lobe of the aedeagus. The median lobe (length: 2.5 mm) is a curved sclerotic tube containing at its convex side an invaginated internal sac (Fig 6A and 6B).

During copulation, this sac becomes evaginated, and the ejaculate is released through the gonopore. The internal sac surface has sclerotic scales which are oriented basally. They are flat at the basal region and become spicule-like towards the gonopore (Fig 6C–6E). Scale shape and orientation are possibly helpful to fix the aedeagus in the female genital tract during copulation. At the concave side of the median lobe small and large parameres are situated. They are surrounded by muscle cells (Fig 6A and 6B).

## Discussion

### Testes

Adephaga (Coleoptera) testes consist of a single tube-like coiled follicle (Lawrence & Britton 1994). Similar to the globular testes of two Sardinian Carabids (Pterostichini) (Carcupino et al. 2002, Cadeddu et al. 2008), *L*. *assimilis* testes contain, in addition, a part of the vas deferens. As by all other insects [37], the complete spermatogenesis proceeds in the follicle. Similar to *P*. *nigrita* [29] and *A*. *parallelpipidus* [19], cyst resolution and transport of single spermatozoa towards the vas deferens appears when spermatogenesis is finished.

### Vasa deferentia

Similar as the spermatic duct of other Carabidae [19] the vas deferens I of *L*. *assimilis* contains diverticula. However, they vary significantly in size and number. *L*. *assimilis* has four times more (~400) diverticula than *Calathus fuscipes* Goeze 1777 (Carabidae: Pterostichini), but they are ca. twice (~160 μm) as long [13]. Similar size ratios are evident when compared to *A*. *parallelpipidus*, but these diverticula appear croissant-like [19]. Similar to *P*. *nigrita* [26], diverticula size increases along the spermatic duct of *L*. *assimilis*, and their ovoid shape is similar to the globular protrusions of *Percus strictus strictus* Dejean 1828 [15].

Probably, similar as that described for other Pterostichini, *A*. *parallelpipidus* [19], *P*. *strictus strictus* [15], *Speomolops sardous* Patrizi 1955 [16], *P*. *nigrita* [14], and *Calathus* species [13], the diverticula containing vas deferens region of *L*. *assimilis* is responsible for sperm bundle formation. Each diverticulum contains one central rod and spermatozoa. We suggest that spermatozoa move from the anterior tract into the diverticulum where they attach at the central rod via their heads. **Ferenz** [14] suggests that there is an active movement mediated by attractants in *P*. *nigrita*. In addition, it could be that the diverticula contractions absorb the spermatozoa of *L*. *assimilis*.

The origin of the substances forming the spermatostyle is not yet clear. Indicated by the HE staining, they are possibly secreted from the diverticulum epithelia cells. The secretory function of diverticulum epithelial cells to produce central rod substances has been described for some Carabidae species [15, 16, 19, 26]. The rhythmic diverticulum contractions could be required to form spermatostyles, as suggested by [26] for *P*. *nigrita*.

The sperm bundle apex of *L*. *assimilis* is not attached by sperm heads, probably because it is not accessible due to its embedding in the apical diverticulum epithelium when spermatozoa attach. Similarly, the spermiozeugmata heads of *P*. *nigrita* are fixed in a microvilli-rich impression of the epithelium in the diverticulum apex [26].

Because sperm bundle formation proceeds after spermatogenesis is completed, *L*. *assimilis* contains secondary sperm bundles, which should be called spermiozeugmata according to **Higginson and Pitnick** [21]. These spermiozeugmata become released from the diverticula and are then transported into the seminal vesicle where an AF-AB staining of spermatozoa indicates the presence of sulphated glycoproteins. In contrast, spermatozoa evident in the spermatic duct region between the proximal follicle and vas deferens I contain, in addition, carboxylated glycoproteins. This indicates a chemical turnover of the structural glycosides in the seminal vesicle whose physiological function is not yet clear.

### Sperm and conjugate morphology

Analysing *L*. *assimilis*, we confirm the spermiozeugmata variability present in the family Carabidae, and for the first time, also within the tribe Platynini. *J*. *estriata*, belonging to the same tribe, shows a similar sperm bundle structure as *L*. *assimilis*, but its spermatostyle is filamentary and 15-times (2.4 mm) longer [18].

Compared to *L*. *assimilis*, species of the related tribe Pterostichini show mostly longer and convoluted sperm bundles [14, 17, 19]. Spermatozoon flagella of this tribe are either freely movable (e.g., in *P*. *strictus strictus*; [15]) or adhered (e.g., in *P*. *nigrita*; [14]). In contrast to *L*. *assimilis*, the spermatozoa of many Pterostichini species are not arranged around the spermatostyle. Instead, they are attached as two strands at both sides of the central rod ([14, 16, 19]. Some Gyrinidae species also have spermatostyles attached by freely moveable spermatozoa [8]. However, the spermatozoon heads of *C*. *insulicola* are only attached at hyaline caps, called spermatodesmata [11, 12].

The AB-PAS staining of the *L*. *assimilis* spermatostyles indicates neutral glycosylated substances. Chemical analyses of Carabidae sperm bundles identified the presence of polysaccharides and proteins [27, 28], suggesting that the central rod of *L*. *assimilis* is also formed by neutral glycoproteins.

Similar to that described for *Membranipora membranacea* Linnaeus 1767 [38], the spiral spermiozeugmata movement of *L*. *assimilis* indicates a coordinated spermatozoa behaviour probably important to improve sperm movability. The number of spermatozoa per conjugate may be positively correlated with swimming velocity, in which a synchronous flagella movement avoids interference [21]. Thus, the ~200 spermatozoa of *L*. *assimilis* arranged in a conjugate seem to improve the velocity compared to that of a single spermatozoon. Due to different osmolarities and the presence of more viscous fluids, the velocity of ~200 μm/min we identified in Ringer solution may be slower than that in the male and female genital tract.

Similar to other Pterygota species, based on SIM imaging we found that the spermatozoa nuclei of *L*. *assimilis*, likely achieved by the substitution of nuclear histones by protamines [39], contain highly condensed chromatin. It is distributed in a lancet-like manner, a spermatozoon head shape common in this insect subclass [1]. The high degree of nuclear chromatin compaction seems to be required to transport effectively the transcriptionally silenced male genetic material into the female egg cells. In addition, the lancet-like head shape may improve the swimming efficiency of the spermatozoa. The variability of sperm nuclei shapes is extraordinary between species [40]. E.g. the worm *Tubiluchus troglodytes* has a double-helical sperm nucleus as identified by electron microscopy [41]. Similarly, the sperm nuclei of *L*. *assimilis* contain a clearly structured, highly condensed reticulate chromatin.

At the end of meiosis, each spermatozoon receives an equal set of mitochondria. They aggregate and form the “nebenkern”, from which two mitochondrial derivates originate [42]. In several hymenopteran species, the two mitochondrial derivates can strongly differ in shape and size [1, 2, 22]. As is typical for Pterygota species [1], and *Cicindela campestris* Linnaeus 1758 (Carabidae: Cicindelinae), [9] and *Pasimachus subsulcatus* Say 1823 (Carabidae: Scaritinae) [10], two parallel mitochondrial derivates occur along nearly the whole flagellum. Interestingly, during the condensation of nuclear chromatin, in *Dendroctonus armandi* Tsai and Li (Coleoptera: Scolytinae), these derivatives acquire different sizes and become laterally located in relation to the nucleus [43]. Similarly, in *Tribolium castaneum* (Herbst, 1797) (Coleoptera: Tenebrionidae) also two asymmetric mitochondrial derivatives were observed [44]. Mature spermatozoa of *Rhynchophorus ferrugineus* Oliv. (Coleoptera: Dryophthoridae) contain mitochondrial derivates of different size, in which the large derivative is embedded into an infolding of the nucleus [45]. In contrast, in *L*. *assimilis* the DNA-specific DAPI staining identified two identical parallel, tightly connected, and recessed mitochondrial derivates, which were found only along the posterior flagellum region. They show a finer reticulated structure, likely representing cristae, than the highly condensed head nucleus chromatin. Similar shaped mitochondrial derivates have not yet been found in other insects. Nevertheless, in despite of the different shape we suggest that they are required for sperm motility as has been found in *Drosophila melanogaster* Meigen, 1830 (Diptera) [46].

### Accessory glands and ejaculatory duct

The accessory glands (a pair of tubes) of *L*. *assimilis* belong to the simplest type known among Coleopterans [25]. This type has also been found in *Leptinotarsa decemlineata* Say 1824 (Coleoptera: Chrysomelidae), *Popillia japonica* Newman 1841 (Coleoptera: Scarabaeidae) and in Cleridae and Carabidae species [30, 47–49]. The accessory glands of *L*. *assimilis* show a curved shape, confirming the finding of **Will et al**. [30]. The gland structure consisting of lumen, columnar epithelium and surrounding muscular layer is typical for many insects [25].

The secretion excreted by the epithelium of many insects mainly contains polysaccharides, proteins and mucosaccharides [31]. The AB-PAS and AF-AB staining suggest that these substances (with exception of non-glycosinolated proteins) also occur in *L*. *assimilis*. Contrary to *L*. *decemlineata* and *P*. *nigrita*, which exclusively include proteins and neutral mucines [48, 50], the gland secretion of *L*. *assimilis* additionally contains acid (carboxylated) mucosubstances.

The main accessory gland function of many insects is the formation of spermatophores [25, 51]. They consist of secretion and spermiozeugmata as found in the bursa copulatrix of female *L*. *assimilis* individuals after copulation [52]. Possibly, in *L*. *assimilis*, the gland lumen secretion is responsible for the formation of spermatophores, similar as is described for *T*. *molitor* (Coleoptera: Tenebrionidae) [53]. However, it is not yet clear where exactly the association of spermiozeugmata and secretion appears because in *L*. *assimilis* glands they were identified only separately. The spermatophore formation could proceed in the posterior (glandular) parts of the vas deferens, similar as has been found by **Krüger et al**. [50] in *P*. *nigrita*.

The circular and longitudinal gland muscle layers of *L*. *assimilis*, present also in *P*. *nigrita* [50] and *S*. *sardous* [16], allow via directed contraction the transport of secretion and spermiozeugmata into the posterior regions of the genital organs. According to **Snodgrass** [24] the region where both accessory glands fuse belongs to the ejaculatory duct. Here, a sclerotic structure is evident, possibly required to stabilize the tract during the transport of lumen content. The sclerotic structure also hints on the ectodermal origin of the ejaculatory duct of *L*. *assimilis* as is present in all other insects [25]. It seems that the *L*. *assimilis* ejaculate contains complete spermiozeugmata because they are still present in the ejaculatory duct, and according to **Fritzsch** [52], they have also been found in the female bursa copulatrix.

### Aedeagus

Similar to other insects, the aedeagus of *L*. *assimilis* is required to transfer the ejaculate into the female during copulation [24]. Although there is high variability its basic structure corresponds to that described by **Sharp and Muir** [54] for Carabids, in which the lobus of *L*. *assimilis* resembles that of *Carabus violaceus* Linnaeus 1758 (Carabidae: Carabinae) [54].

The internal sac of many insects shows scales and spikes [24]. That of *L*. *assimilis* has sclerotic scales, but no additional spikes as is found in *P*. *nigrita* [55], which are probably essential for its fixation in the female bursa copulatrix. As suggested for other insects [24], the parameres of *L*. *assimilis* seem to serve as brace organs during copulation. Due to an increased pressure caused by haemolymph during the in vivo copulation [56], the internal sac should be larger than is visible in the preparation shown in Fig 6B.

## Supporting information

S1 Fig Two parallel mitochondrial derivates are twisted around each other in the flagellum. (TIF)

S2 Fig Structures of the posterior follicle, vas efferens and vas deferens region I. (TIF)

S3 Fig Lateral cross section of the area where the accessory glands lead into the ejaculatory duct stained by HE. (TIF)

S1 Table Size of the different organs of the reproductive system and the spermatozoa. (XLSX)

S1 Movie Flagellum region free of mitochondrial derivates, and sperm head stained by DAPI. (MP4)

S2 Movie Condensed reticulate sperm head chromatin interspersed by DNA-free regions identified by SIM. (MP4)

S3 Movie Mitochondrial derivates along the posterior flagellum region showing constrictions and swells. (MP4)

S4 Movie Mitochondrial derivates showing a tiny gap between them (arrow). (MP4)

S5 Movie Diverticula contraction (arrow). (MP4)

S6 Movie Spermiozeugma movement induced by coordinated flagella fluctuations. The real time recording reflects a duration of 28 seconds.
(MP4)

## References

[1] Jamieson BGM. The ultrastructure and phylogeny of insect spermatozoa. Cambridge University Press, Cambridge, 1987; 319 pp.

[2] Dallai R. Overview on spermatogenesis and sperm structure of Hexapoda. Arthropod Structure and Development 2014; 43:257–90. doi: 10.1016/j.asd.2014.04.002 24732045 10.1016/j.asd.2014.04.002

[3] Pitnick S, Hosken DJ, Birkhead TR. Sperm morphological diversity In: Birkhead TR et al (eds) Sperm biology: an evolutionary perspective. Academic Press, Amsterdam 2009; 69–149.

[4] Gilson G. Etude compare´e de la spermatoge´ne`se chezles arthropods. La Cellule 1884; 1:84–94.

[5] Ballowitz E. Spermiozeugmen bei Libellen. Biologisches Zentralblatt 1916; 36:209–16.

[6] Bawa SR, Marwaha RK. The sperm bundles of honeybee *Apis cerana indica* Fabr. Experientia. 1974; 31:684–686.

[7] Viscuso R, Narcisi L, Sottile L, Barone N. Structure of spermatodesms of *Orthoptera tettigonioidea*. Tissue Cell 1998; 30:453–463. 18627847 10.1016/s0040-8166(98)80059-1

[8] Breland OP, Simmons E. Preliminary studies of the spermatozoa and the male reproductive system of some whirligig beetles (Coleoptera: Gyrinidae). Entomology News 1970; 81:101–110.

[9] Werner G. Untersuchungen über die Spermiogenese beim Sandläufer *Cicindela campestris* L. Zeitschrift für Zellforschung und Mikroskopische Anatomie 1965; 66: 255–275. 14311515

[10] Witz BW. Comparative ultrastructural analysis of spermatogenesis in *Pasimachus subsulcatus* and *P*. *strenuus* (Coleoptera, Carabidae). Invertebrate Reproduction and Development 1990; 18:197–203.

[11] Takami Y. Mating behavior, insemination and sperm transfer in the ground beetle *Carabus insulicola*. Zoological Science 2002; 19:1067–1073. doi: 10.2108/zsj.19.1067 12362062 10.2108/zsj.19.1067

[12] Takami Y, Sota T. Sperm competition promotes diversity of sperm bundles in Ohomopterus ground beetles. Die Naturwissenschaften 2007; 94:543–550. doi: 10.1007/s00114-007-0225-3 17318611 10.1007/s00114-007-0225-3

[13] Gilbert O. The natural histories of four species of *Calathus* (Coleptera: Carabidae) living on sand dunes in Anglesey, North Wales. Oikos 1956; 7:22–47.

[14] Ferenz H-J. Structure and formation of sperm bundles in the carabid beetle *Pterostichus nigrita* In: Boer Den et al. (eds.) Carabid beetles—their adaptations and dynamics. Gustav Fischer Verlag, Stuttgart 1986; 147–155.

[15] Carcupino M, Stocchino AG, Corso G, Manca I, Casale A. Morphology of the male reproductive apparatus and spermatodesms formation in Percus strictus strictus (Coleoptera, Carabidae). 9th International Symposium of Spermatology 2002; 31–34.

[16] Cadeddu B, Casale A, Marcia P, Stocchino AG. Aspetti morfologici e funzionali dell’ apparato riproduttore di Speomolops sardous (Coleoptera, Carabidae) In: Marcia P (ed) Strategie per il monitoraggio e la conservazione della fauna ipogea in alcuni siti della Sardegna. University Sassari, 2008.

[17] Sasakawa K. Sperm bundle and reproductive organs of carabid beetles tribe *Pterostichini* (Coleoptera: Carabidae). Die Naturwissenschaften 2007; 94:384–91. doi: 10.1007/s00114-006-0200-4 17165077 10.1007/s00114-006-0200-4

[18] Sasakawa K, Toki W. A new record, sperm bundle morphology and preliminary data on the breeding type of the ground beetle *Jujiroa estriata* Sasakawa (Coleoptera: Carabidae: Platynini). Entomological Science 2008; 11:415–417.

[19] Löser S, Lampe K, H. Die Morphologie und Histologie der Vasa deferentia von Abax ater VILL. (Coleoptera: Carabidae) und die in ihm stattfindende Spermiozeugmenbildung. Verhandlungen der Deutschen Zoologischen Gesellschaft, 66. Jahresversammlung, Gustav Fischer Verlag 1973; 83–87.

[20] Hayashi F. Sperm-cooperation in the fish-fly, *Parachaulioides japonicus*. Functional Ecology 1998; 12:347–350.

[21] Higginson DM, Pitnick S. Evolution of intra-ejaculate sperm interactions: do sperm cooperate? Biological Reviews of the Cambridge Philosophical Society 2011; 86:249–270. doi: 10.1111/j.1469-185X.2010.00147.x 20608927 10.1111/j.1469-185X.2010.00147.x

[22] Phillips DM. Insect sperm: their structure and morphogenesis. The Journal of Cell Biology 1970; 44:243–277. 4903810 10.1083/jcb.44.2.243 PMC2107952

[23] Lawrence JF, Britton EB. Australian beetles. Melbourne University Press 1994; x+192 pp.

[24] Snodgrass RE. Principles of insect morphology. Cornell University Press, Ithaca 1993; pp 550–623.

[25] Kaulenas MS. Insect accessory reproductive structures: Function, structure and development: Springer Verlag, Berlin 1992; pp 3–12 and 123–150.

[26] Hodgson AN, Ferenz HJ, Schneider S. Formation of sperm bundles in *Pterostichus nigrita* (Coleoptera: Carabidae). Invertebrate Reproduction and Development 2013; 57:120–131.

[27] Mackie JB, Walker MH. A study of the conjugate sperm of the dytiscid water beetles *Dytiscus marginalis* and *Colymbetes fuscus*. Cell and Tissue Research 1974; 148:505–519. 4836644 10.1007/BF00221935

[28] Hayashi F. A trypsin-degradable protein agglutinates fish-fly sperm-bundles (Megaloptera: Corydalidae). International Journal of Insect Morphology and Embryology 1997; 26:63–66.

[29] Wölfer M. Untersuchungen zur Spermiozeugmen-Bildung beim Laufkäfer Pterostichus nigrita (Paykull 1790): Diploma Thesis, Martin-Luther-University Halle-Wittenberg 2010; 31–36.

[30] Will KW, Liebherr JK, Maddison DR, Galian J. Absence asymmetry: the evolution of monorchid beetles (Insecta: Coleoptera: Carabidae). Journal of Morphology 2005; 264:75–93. doi: 10.1002/jmor.10319 15732050 10.1002/jmor.10319

[31] Gillott C. Male accessory gland secretions: modulators of female reproductive physiology and behavior. Annual Review of Entomology 2003; 48:163–84. doi: 10.1146/annurev.ento.48.091801.112657 12208817 10.1146/annurev.ento.48.091801.112657

[32] Freude H, Harde KW, Lohse GA. Die Käfer Mitteleuropas Volume 2 Adephaga 1, Goecke and Evers, Krefeld 1976; 223 pp.

[33] Romeis B. Mikroskopische Technik Mulisch M, Welsch U (eds), 18th edition, Spektrum Akademischer Verlag, Heidelberg 2010; 214–215.

[34] Spicer SS, Meyer DB. Histochemical differentiation of acid mucopolysaccharides by means of combined aldehyde fuchsin-alcian blue staining. Technical Bulletin of the Registry of Medical Technologists 1960; 30:53–60. 13833304

[35] Weisshart K, Fuchs J, Schubert V. Structured Illumination Microscopy (SIM) and Photoactivated Localization Microscopy (PALM) to Analyze the Abundance and Distribution of RNA Polymerase II Molecules in Flow-Sorted Arabidopsis Nuclei. Bio-protocol 2016; 6(3): e1725, http://wwwbio-protocolorg/e1725.

[36] Bock C. A quick and simple method for preparing soft insect tissues for scanning electron microscopy using carnoy and hexamethyldisilazane. Beiträge zur Elektronenmikroskopie und Direktabbildung von Oberflächen 1987; 20:209–214.

[37] Dettner K, Peters W. Lehrbuch der Entomologie. 2nd edition, Gustav Fischer Verlag, Heidelberg 2010.

[38] Temkin MH, Bortolami SB. Waveform dynamics of spermatozeugmata during the transfer from paternal to maternal individuals of *Membranipora membranacea*. Biological Bulletin 2004; 206:35–45. doi: 10.2307/1543196 14977728 10.2307/1543196

[39] Hecht NB. The making of a spermatozoon: a molecular perspective. Developmental Genetics 1995; 16:95–103. doi: 10.1002/dvg.1020160202 7736670 10.1002/dvg.1020160202

[40] Skinner BM, Johnson EEP. Nuclear morphologies: their diversity and functional relevance. Chromosoma 2017; 126:195–212. doi: 10.1007/s00412-016-0614-5 27631793 10.1007/s00412-016-0614-5 PMC5371643

[41] Ferraguti M, Garbelli C. The spermatozoon of a 'living fossil': *Tubiluchus troglodytes* (Priapulida). Tissue and Cell. 2006; 38:1–6. doi: 10.1016/j.tice.2005.05.001 16274719 10.1016/j.tice.2005.05.001

[42] Pratt SA. An electron microscope study of nebenkern formation and differentiation in spermatids of *Murgantia histrionica* (Hemiptera, Pentatomidae). Journal of Morphology 1968; 126:31–66. doi: 10.1002/jmor.1051260104 5697117 10.1002/jmor.1051260104

[43] Wu YF, Wei LS, Anthony Torres M, Zhang X, Wu SP, Chen H. Morphology of the male reproductive system and spermiogenesis of *Dendroctonus armandi* Tsai and Li (Coleoptera: Curculionidae: Scolytinae). Journal of Insect Science 2017; 17:1–9. 28130461 10.1093/jisesa/iew116 PMC5270412

[44] Dias G, Lino-Neto J, Mercati D, Dallai R. The sperm ultrastructure and spermiogenesis of *Tribolium castaneum* (Coleoptera: Tenebrionidae) with evidence of cyst degeneration. Micron 2015; 73:21–27. doi: 10.1016/j.micron.2015.03.003 25867758 10.1016/j.micron.2015.03.003

[45] Paoli F, Dallai R, Cristofaro M, Arnone S, Francardi V, Roversi PF. Morphology of the male reproductive system, sperm ultrastructure and gamma-irradiation of the red palm weevil *Rhynchophorus ferrugineus* Oliv. (Coleoptera: Dryophthoridae). Tissue and Cell 2014; 46:274–285. doi: 10.1016/j.tice.2014.06.003 25015762 10.1016/j.tice.2014.06.003

[46] Hales KG, Fuller MT. Developmentally regulated mitochondrial fusion mediated by a conserved, novel, predicted GTPase. Cell 1997; 90:121–129. 9230308 10.1016/s0092-8674(00)80319-0

[47] Anderson JM. A cytological and cytochemical study of the male accessory reproductive glands in the japanese beetle, *Popillia-Japonica* Newman. Biological Bulletin 1950; 99:49–64. doi: 10.2307/1538751 14772244 10.2307/1538751

[48] De Loof A, Lagasse A. The ultrastructure of the male accessory reproductive glands of the colorado beetle. Zeitschrift für Zellforschung und Mikroskopische Anatomie 1972; 130:545–552. 4263606 10.1007/BF00307006

[49] Opitz W. Spermatophores and spermatophore producing internal organs of *Cleridae* (Coleoptera: Clerinae): Their biological and phylogenetic implications. Coleopterists Bulletin 2003; 57:167–190.

[50] Krüger S, Ferenz HJ, Randall M, Hodgson AN. Structure of the male reproductive accessory glands of *Pterostichus nigrita* (Coleoptera: Carabidae), their role in spermatophore formation. Invertebrate Reproduction and Development 2014; 58:75–88.

[51] Chen PS. The functional morphology and biochemistry of insect male accessory glands and their secretions. Annual Review of Entomology 1984; 29:233–255.

[52] Fritzsch D. Morphologie der inneren Reproduktionsorgane von weiblichen Limodromus assimilis (Paykull, 1790) (Coleoptera: Carabidae). Bachelor Thesis, Martin-Luther-University Halle-Wittenberg 2013; pp 23–25.

[53] Happ GM. Maturation of the male reproductive system and its endocrine regulation. Annual Review of Entomology 1992; 37:303–320. doi: 10.1146/annurev.en.37.010192.001511 1539938 10.1146/annurev.en.37.010192.001511

[54] Sharp D, Muir F. The comparatative anatomy of the male genital tube in Coleoptera. Transactions of the Entomological Society of London 1912; 60:477–642.

[55] Koch D. Morphological-physiological studies on *Pterostichus nigrita* (Coleoptera: Carabidae), a complex sibling species In: Den Boer et al (eds), Carabid beetles—their adaptations and dynamics. Gustav Fischer Verlag, Stuttgart 1986; 267–279.

[56] Ishikawa R. Notes on some basic problems in the taxonomy and the phylogeny of the subtribe *Carabina* (Coleoptera: Carabidae). Bulletin of the National Science Museum 1973; 16:191–215.

