# Supplementary figures and images for: Male reproductive system and spermatogenesis of Limodromus assimilis (Paykull 1790)
Source: PLoS One. 2017 Jul 19;12(7):e0180492. doi: 10.1371/journal.pone.0180492 (PMC5516968; doi:10.1371/journal.pone.0180492)

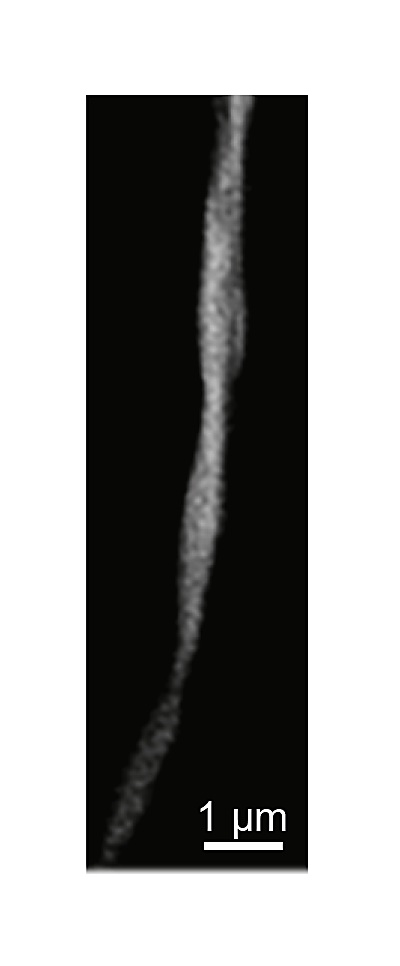

Supplement: S1 Fig — (TIF) [file pone.0180492.s001.tif]

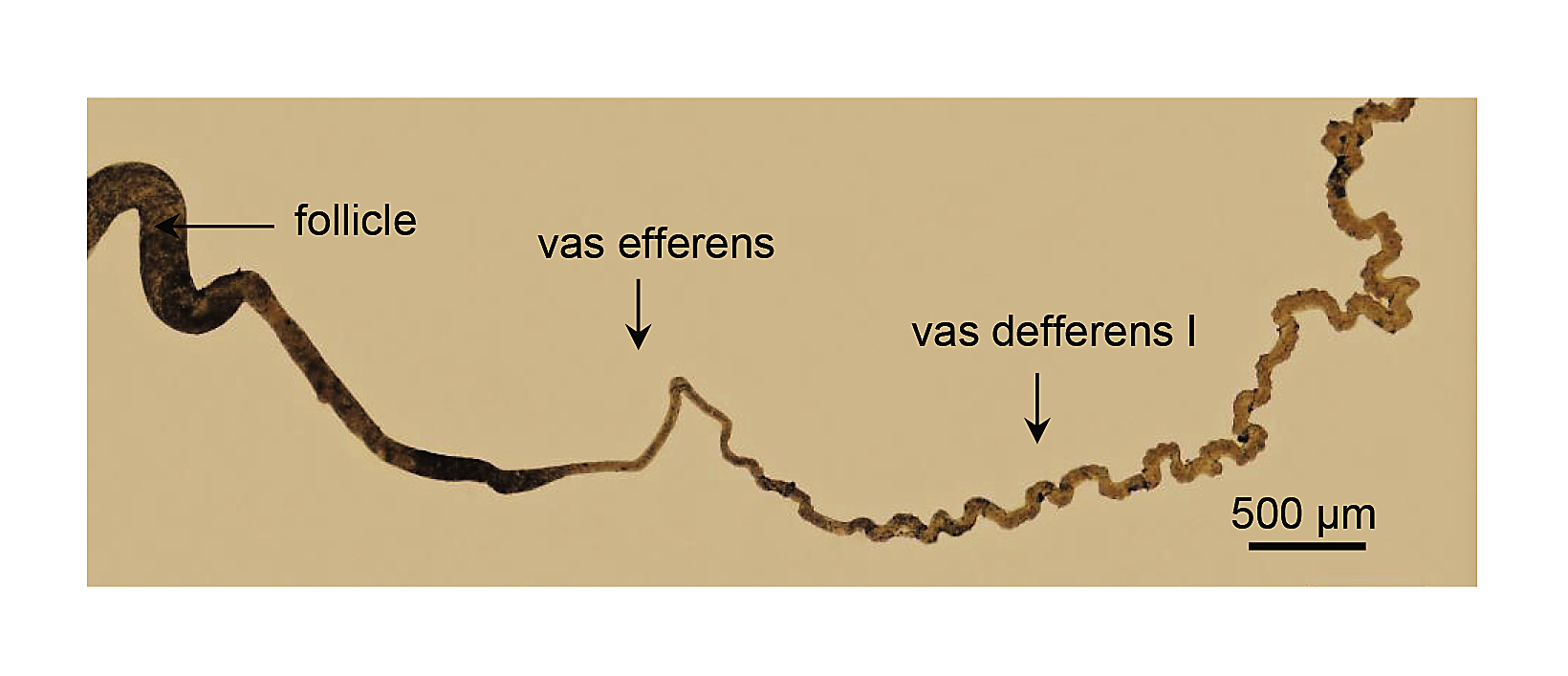

Supplement: S2 Fig — (TIF) [file pone.0180492.s002.tif]

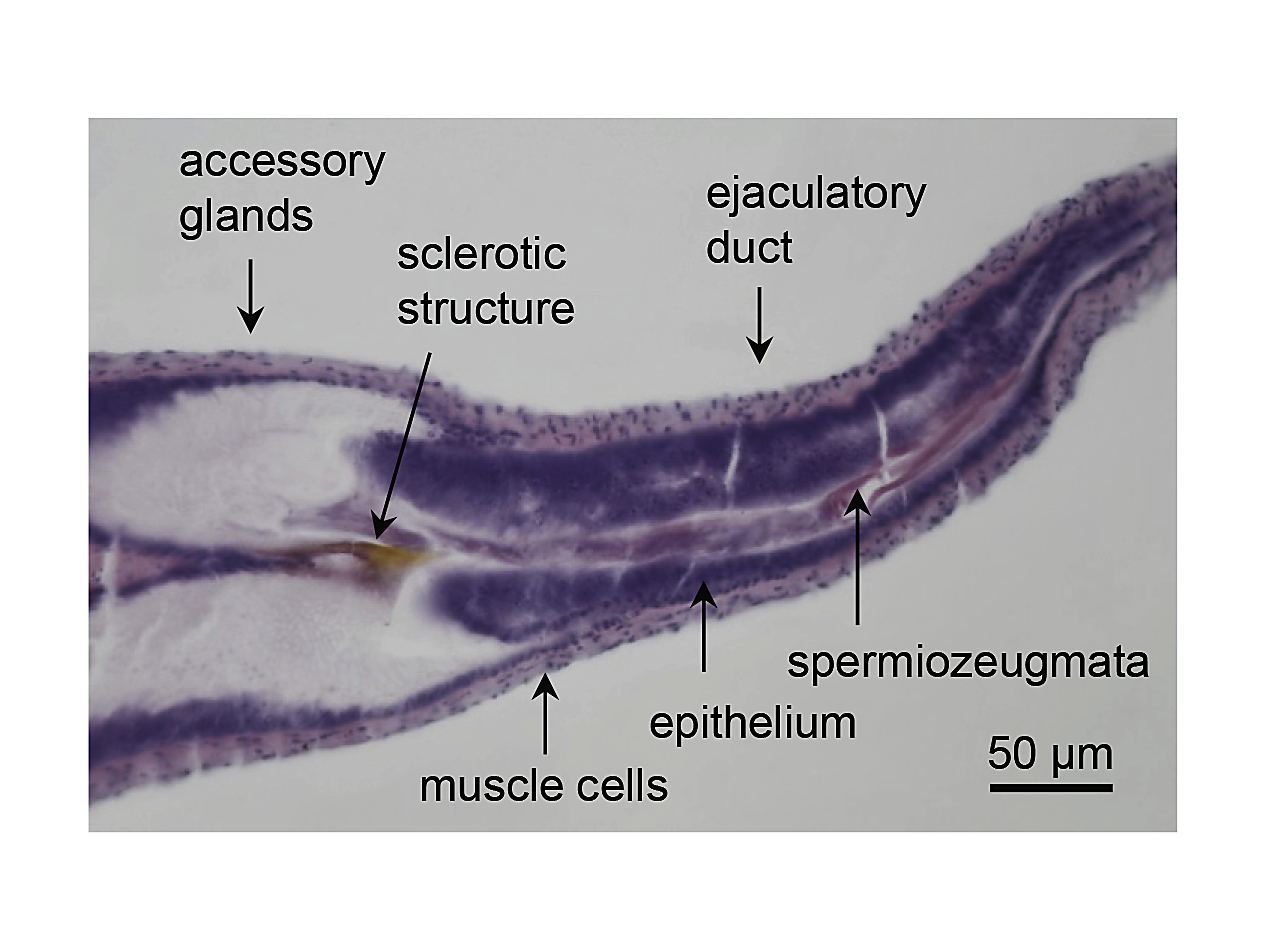

Supplement: S3 Fig — (TIF) [file pone.0180492.s003.tif]
